# Deletion in *CACNA1F* gene causes X-linked progressive retinal atrophy in English Cocker Spaniel dogs

**DOI:** 10.1186/s12917-026-05421-y

**Published:** 2026-03-25

**Authors:** A. Bionda, Luigi Liotta, V. Floridia, J. K. Niggel, D. Giretto, P. Crepaldi, G. D. Aguirre, L. Murgiano

**Affiliations:** 1 Department of Agricultural and Environmental Sciences—Production, Landscape, Agroenergy, University of Milan, Milan, 20133 Italy; 2 Department of Veterinary Sciences, University of Messina, Messina, 98168 Italy; 3 Division of Experimental Retinal Therapies, Department of Clinical Sciences & Advanced Medicine, University of Pennsylvania, Philadelphia, 19104 USA; 4 Sylvia M. Van Sloun Laboratory for Canine Genomic Analysis, University of Pennsylvania, Philadelphia, 19104 USA; 5 Centro Medico Veterinario Cartesio, Melzo, 20066 Italy

**Keywords:** Progressive retinal atrophy, Dog, *CACNA1F*, X-linked, English Cocker Spaniel

## Abstract

**Background:**

Progressive retinal atrophy (PRA) was diagnosed in four related male English Cocker Spaniels (ECS) between the ages of 3 and 4 years. All affected dogs were born to clinically unaffected parents that had tested negative for known PRA-causing variants through commercial vendors. Pedigree analysis revealed that the mothers of the affected dogs were paternal half-siblings, and their father had been reported as visually impaired, suggesting an X-linked recessive mode of inheritance.

**Results:**

To identify the underlying genetic cause, we performed homozygosity mapping, identifying six candidate regions, and whole-genome sequencing. A single high-impact coding variant—a 1-bp deletion in exon 36 of the *CACNA1F* gene on X chromosome (UU_Cfam_GSD_1.0 NC_049260.1 g.42,516,353del) —was identified. This variant is predicted to cause a frameshift and premature stop codon (XM_038587436.1, c.4,481del, XP_038443364.1:Phe1482LeufsTer8) and was found in a hemizygous state in all affected males and heterozygous in their mothers, while being absent in fathers as well as unaffected control dogs. A specifically designed PCR test was developed and applied to a broader cohort of 92 related and unrelated ECS dogs, confirming the segregation of this variant.

**Conclusions:**

This study identified a novel segregating *CACNA1F* variant, likely to be responsible for an X-linked recessive form of PRA in the ECS. Given that *CACNA1F* is already associated with retinal disorders in other mammals, including humans, this finding adds to its relevance across species. This study highlights the importance for breeders of regular health examinations for late-onset conditions and prompt reporting of hereditary disorders of unknown cause to enable rapid identification of their genetic basis. Development and implementation of specific genetic screening tests are recommended to assess the frequency of this variant in ECS and other populations, inform breeding strategies, and prevent further dissemination of this deleterious variant.

**Supplementary Information:**

The online version contains supplementary material available at 10.1186/s12917-026-05421-y.

## Introduction

 Progressive Retinal Atrophy (PRA) refers to a group of inherited retinal diseases characterized by bilateral progressive photoreceptor degeneration, ultimately leading to vision loss. These conditions are genetically and clinically similar to human retinitis pigmentosa (RP), as both are characterized by the degeneration of photoreceptors, initially in rods and followed at variable intervals by cones [1–3].

Although the similarities between these human and canine diseases have been recognized since the mid-20th century [4], the first genetic variant linked to PRA, involving the *PDE6B* gene, was identified only in the late 1980s in the Irish Setter breed (OMIA:000882–9615) [5]. Since then, over 31 variants in 24 genes have been linked to canine retinal diseases, most of which follow an autosomal recessive inheritance pattern [6]. Among these, a unique c.5G > A variant in the *PRCD* (Photoreceptor Disc Component) gene (OMIA:001298–9615), also found in some RP patients, is the most widespread form of PRA, affecting more than 50 dog breeds, suggesting that it likely originated from an ancient founder [7]. In addition to autosomal recessive forms, autosomal dominant [8] and X-linked forms of PRA (XLPRA) also exist in both dogs and humans. In dogs, two main XLPRA forms, both being caused by deletions in exon ORF 15 of the *RPGR* (Retinitis Pigmentosa GTPase Regulator) gene have been described: XLPRA1, found in Siberian Huskies, Samoyeds, and Weimaraners [9–11], and XLPRA2, identified initially in mix breed dogs [11] (OMIA:000831–9615, OMIA:001518–9615, OMIA:001432–9615).

Clinically, PRA usually presents first as night blindness (nyctalopia) and gradually progresses to complete vision loss. On ophthalmologic examination, characteristic changes include tapetal hyperreflectivity, attenuation of superficial retinal blood vessels, and secondary atrophy of the optic nerve head [12]. Functional assessment with electroretinography (ERG) typically shows a marked reduction in photoreceptor activity, with rod dysfunction occurring earlier and more severely than cone impairment [11, 13–15].

To date, more than 100 dog breeds and crossbreeds are known to be affected by various PRA forms [5]. While clinical symptoms are consistent across PRA forms, the age of onset and disease progression rates vary significantly among breeds and variants, and even in breeds with the same variant there is also phenotype variation [1, 15, 16].

The English Cocker Spaniel (ECS) is particularly predisposed to PRA, with most cases attributable to the *PRCD* c.5G > A genetic variant (OMIA:001298–9615) [15, 17]. Several studies report allele frequencies ranging from 7% to 34% across different countries [18–21], with a recent investigation of 77 Italian ECS reporting a frequency of 14% [22]. However, some studies comparing genetic test results with clinical diagnoses have noted that not all clinically affected ECS were homozygous for the *PRCD* variant [23], suggesting that other genetic variants contribute to the disease in this breed [19]. In addition to the *PRCD* variant (9.5% prevalence), a study investigating a large multi-breed cohort of dogs identified another variant (OMIA:001432–9615) in the *RPGRIP1* (RPGR Interacting Protein 1) gene responsible for cone-rod dystrophy in the 579 analyzed ECS [19].

The identification of unknown variants responsible for PRA and the development of genetic tests are of critical importance, both for the prevention of inherited blinding diseases through use of DNA testing, and for development of therapeutic options for this disease which are currently limited. The present study aims to investigate the genetic variation underlying a novel form of PRA in the ECS breed, which was brought to our attention by an Italian breeder who observed cases of PRA in dogs born to parents that had tested negative for PRCD-PRA and other known retinal disease variants.

## Materials and methods

### Ethical statement

Concerning the USA dogs, research and examinations were conducted in full compliance with the Association for Research in Vision and Ophthalmology (ARVO) “Resolution on the Use of Animals in Ophthalmic and Vision Research”. This protocol was approved by the “Institutional Animal Care and Use Committee” (IACUCs, University of Pennsylvania #806301). Ethical approval was not required for Italian dogs, as ophthalmological health data were obtained from voluntary examinations conducted independently by owners and breeders. Genetic analyses were performed using biological samples previously deposited with the Italian Kennel Club (ENCI) in accordance with the technical standards of the Italian genealogical register. No procedures were explicitly conducted for research purposes, and no animals were handled or sampled directly by the authors.

### Phenotype assessment

**Fig. 1:**
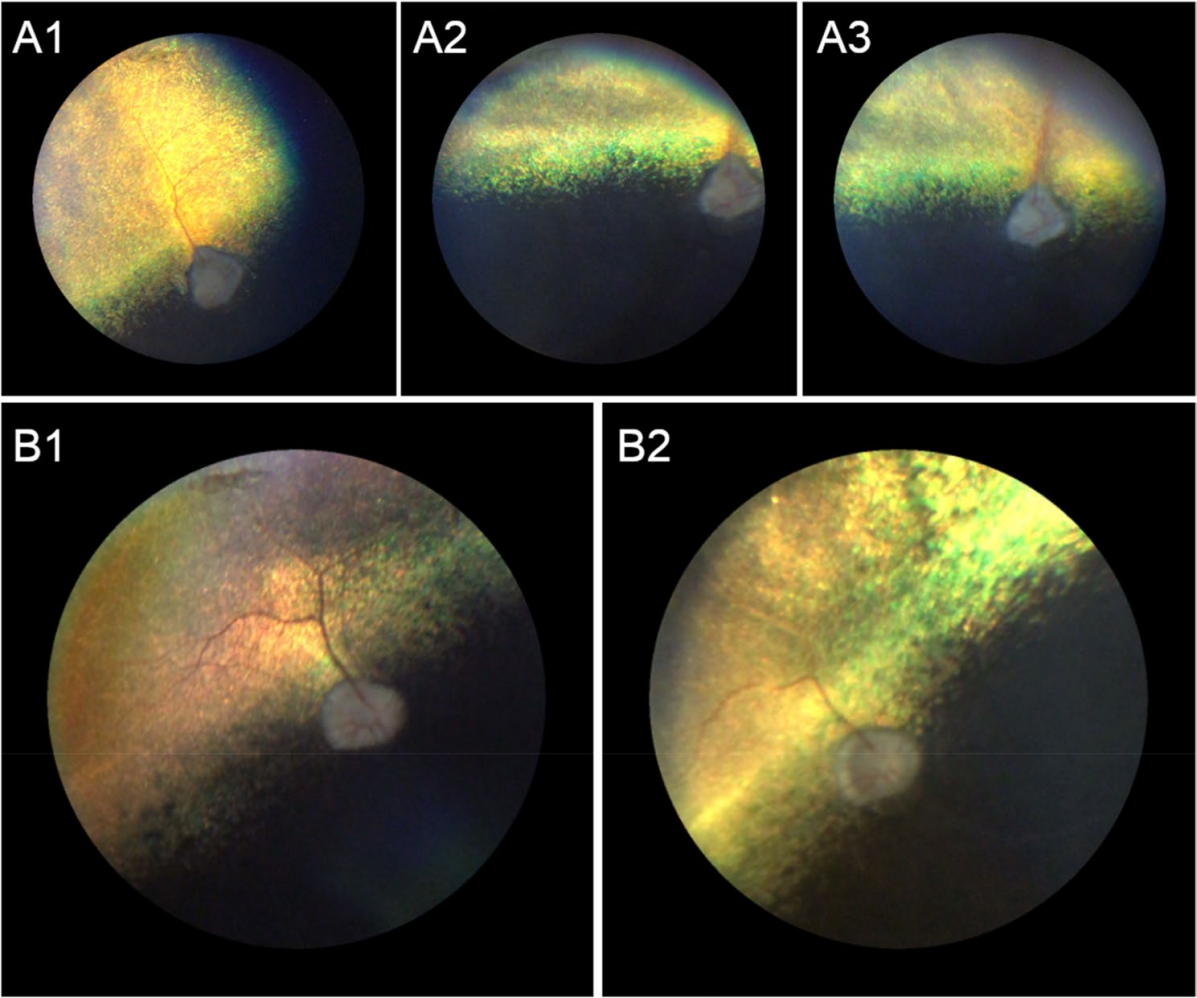
Retinal phenotype. Fundus photographs taken from littermate affected English Cocker Spaniels ECS86 (OS: **A1**-**3**) and ECS87 (OD: **B1**,**2**). The fundus images were taken when dogs were 3.5 years (**A1**, **B1**) and 4.3 years (**A2**-**3**, **B2**). The photographs show characteristic findings of late-stage retinal degeneration: tapetal hyperreflectivity, vessel attenuation (more prominent in ECS86) and optic disc pallor. OD=right eye; OS=left eye

Dogs included in the study were examined by an ECVO board-certified veterinary ophthalmologist or veterinarians experienced in clinical ophthalmological examinations and disease assessment. Examinations were made using a mydriatic for pupillary dilatation, biomicroscopy and indirect ophthalmoscopy. In selected cases, fundus photography was carried out to better document the clinical observations (Fig. 1). Most of the study dogs came from the same breeder and are related. Initially, they were examined because some of the dogs were showing different degrees of vision impairment, and not as part of a routine eye health program to screen for ocular disorders that were heritable. Once a diagnosis was made on a subset of dogs, pedigree analysis was used to identify other potential affected dogs, and clinical examinations in a larger sample size were carried out.

### Sample collection

Genomic DNA derived from blood samples from a total of 95 ECS (including 4 affected, 11 unaffected siblings or half-siblings, 4 unaffected parents, and 3 ancestors) was used for the study. 51 of these dogs were from Europe (Italy), including all cases and relatives, and all their pedigrees were retrieved from ENCI. In particular, the initial samples of Italian dogs were provided by the owners of the affected subjects, while additional samples from relatives traced through pedigree analysis were provided by the accredited ENCI laboratories, where the blood had been stored in accordance with the Italian studbook regulations. Additional Northern American ECS controls were provided by the laboratory at the University of Pennsylvania. Specifically, internal controls publicly available are part of bioproject PRJNA937381 (File S1). DNA was extracted with the Illustra DNA extraction kit BACC2 (GE Healthcare), following the manufacturer’s instructions.

### Mapping of the causative variant

#### Genotyping and homozygosity mapping

**Fig. 2:**
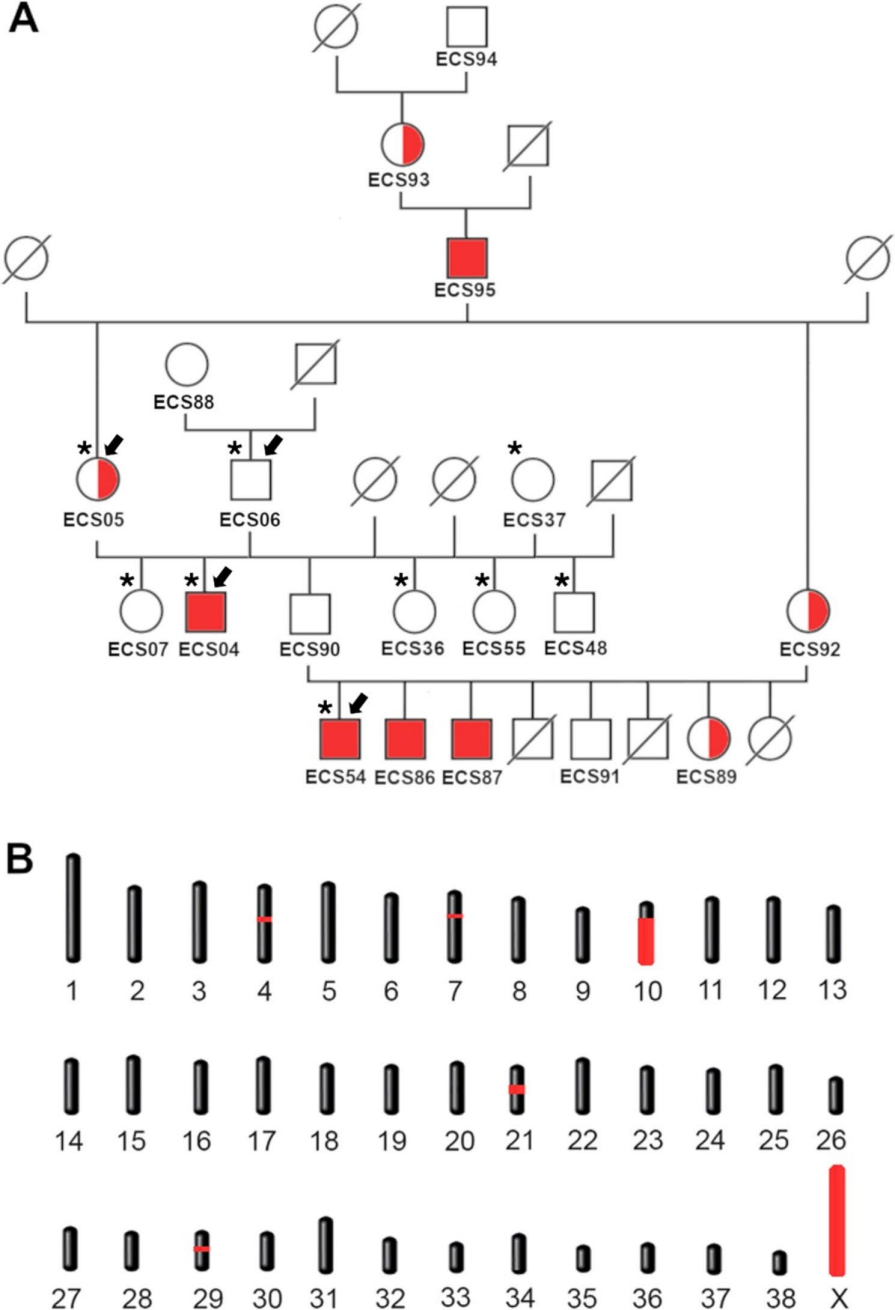
Family tree and mapping of the candidate regions. **A** Family tree of the cases. The four hemizygous cases (ECS04, ECS54, ECS86, and ECS87) and their hemizygous ancestor (ECS95) are shown with a red-filled shape. Genotyped female carriers (ECS05, ECS89, ECS92, and ECS93) and wild-type controls are shown with a half-filled and empty shape, respectively. Males are indicated with a square, females with a circle. Dogs for which there is no available sample are indicated with a diagonal bar. It is to be noted that the common ancestor of the cases’ mothers (ECS95) was reported to be visually impaired. Whole-genome sequenced probands are indicated with arrows, and SNP-chip genotyped dogs are indicated with asterisks. **B** Mapping of the candidate regions. In red, highlighted the autosomal homozygous regions exclusive for the cases and chromosome X

Genotyping was carried out on 9 ECS (2 cases initially detected, 2 unaffected parents, plus 5 other related unaffected ECS, marked with an asterisk in Fig. 2A) on a 220k Illumina canine SNP chip following the standard protocols recommended by the manufacturer.

Homozygosity mapping of the 9 genotyped dogs was carried out with PLINK v.1.9 [24] to detect extended homozygosity intervals with shared alleles. We used the commands --merge (multiple batches of genotyped dogs) and --geno 0.1 to retain 211,122 markers after filtering. Homozygosity analysis was carried out using “--homozyg” and “--homozyg group”, standard parameters. Since we could not exclude an X-linked inheritance, the whole CFAX was automatically included as a candidate region. CanFam3.1 coordinates were converted to canFam4 using the UCSC LiftOver remapping service (https://genome.ucsc.edu/cgi-bin/hgLiftOver, accessed 27 January 2026). 

#### Whole-genome sequencing

For the two cases described and the two parents of one of the cases (indicated with arrows in Fig. 2A), the PCR-free library was prepared as follows. Libraries of 300 bp insert size were prepared and sequenced in Illumina NovaSeq6000 (paired-end reads, 2 × 100 bp). Fastq files were generated using Casava 1.8: a total of 1,500M reads (100 bp paired-end reads) were collected and successfully mapped for the sequenced dogs (corresponding to an approximate average 30× coverage of the genome for the cases, and 22x for the parents). The paired-end reads were mapped against the dog reference genome UU_Cfam_GSD_1.0 (canFam4); the BAM files were processed, as previously described [25]. 

#### SNV and short indel detection

GATK (version 4.2.3.0 ) [26] was employed for variant calling, using the “HaplotypeCaller” as previously described [25], and for the functional impact of the detected variants, SnpEff v 5.4 was used [27]. Candidate variants mapped in the candidate intervals (homozygous autosomal or chromosome X) were filtered against the Dog10k [28], which contained three ECS. Further searching was conducted in the European Variation Archive variant browser (https://www.ebi.ac.uk/eva/?Variant-Browser, accessed 9 August 2025).

#### Structural variants discovery

Delly2 [29] was used to identify Duplications, Inversions, Translocations, Insertions, and Deletions. BAM files from unrelated dogs belonging to other studies carried out either by our group or from the Dog10k database [30, 31] were used as controls. The commands for deletions, insertions, inversion, translocations, and duplications were all executed separately and for each dog of the sequenced quartet. Each of these analyses was carried out, focusing on the candidate region identified by mapping.

#### Investigation of the Exon 36 1-bp deletion and validation by sanger sequencing

The *CACNA1F* (Calcium Voltage-Gated Channel Subunit Alpha1 F) 1-bp deletion was re-sequenced in the cases and available controls by targeted PCR followed by Sanger sequencing. PCR primers were designed using PRIMER3 [32]. PCR products were amplified using primers flanking the exon 36 1-bp deletion, F (5′- TGTGTGTGGTGGTGGACAG-3′ chrX:42,516,252-42,516,270 and R (5′-GACTCTACCAGCTCAGCAC-3′ chrX:42,516,485-42,516,503). All the coordinates reported here are canFam4. PCR was carried out with AmpliTaqGold360MasterMix (Life Technologies, Carlsbad, CA, USA).

PCR products were run on 1.2% agarose gel, 0.5 µg/mL ethidium bromide. The wild-type amplicon spans 252 bp (chrX 42,516,252 to 42,516,503). Sanger electropherograms were visualized with 4Peaks (https://nucleobytes.com/4peaks/, accessed 8 September 2025).

## Results

### Phenotype characterization

Two male ECS dogs (ECS86 and ECS87) were brought to our attention due to showing signs of a progressive onset of vision deficits. Retinal signs were first detected at approximately 3-4 years of age (Fig. 1). Based on the advanced stage of disease found at 3.5 years of age, it is very likely that the abnormalities initiated much earlier. The cases were part of the same litter, and another littermate (ECS54) displayed similar clinical signs (Fig. 2A). Their parents were reported as clinically normal and clear for PRCD variant. A subsequent examination 9 months later showed that late-stage disease progressed slightly, if at all. At such late disease stages, it is difficult to assess progression as the rate of cell loss slows when the Outer Nuclear Layer (ONL) is reduced to 1-2 nuclei in thickness [33, 34].

Information about the ophthalmologic examination of ECS92 (the mother of the affected dogs ECS86, ECS87, and ECS54) was obtained from the owner, who provided certificates from annual ophthalmologic examinations performed by a certified veterinary specialist. These examinations were conducted to detect and formally certify the absence of congenital and hereditary ocular and retinal diseases. All evaluations consistently confirmed that the ECS92 was free from any alteration indicative of hereditary retinopathies. Furthermore, the medical records contained no indication that patchy retinal degeneration was either observed or suspected.

### Samples and pedigree reconstruction

Blood samples were collected from the two examined cases, related dogs, and other subjects displaying retinal atrophy (Fig. 2A). Specifically, we obtained blood samples from the case ECS04, its unaffected parents (ECS05 and ECS06), an unaffected paternal grandmother (ECS88), and a littermate (ECS07) deemed unaffected after eye exam by a board-certified veterinary ophthalmologist. Additionally, we took samples from the case ECS54 as well as two affected (ECS86 and ECS87, both males) and two unaffected (female ECS89 and male ECS91) littermates, and the parents of this litter (ECS90 and ECS92). Finally, the maternal grandfather of all cases (ECS95, i.e., father of ECS05 and ECS92), his mother (ECS93), and his maternal grandfather (ECS94, i.e., father of ECS93) were sampled. We then gathered 36 unaffected Italian dogs born or used as breeders within the same kennel and related to the cases at different degrees (File S2, extended pedigree) and 44 unrelated unaffected subjects from the USA, and five unrelated individuals displaying PRA symptoms. However, the latter were genotyped as homozygous for the known *PRCD* PRA-causing variant (c.5G>A) and consequently excluded because the *PRCD* known variant already explained the genetic aetiology. Notably, all the other enrolled dogs, including the cases, did not carry the *PRCD* variant.

Therefore, a total of four cases were examined. Information gathered about the immediate family of the cases revealed unaffected parents. The reconstructed pedigree information allowed us to identify a common male ancestor, i.e. the maternal grandfather of all the cases, who was, interestingly, reported to be visually impaired (Fig. 2A). For this reason, the immediate family information suggested a possible autosomal recessive mode of inheritance. As the parents of the unaffected dogs were phenotypically normal, we considered a fully penetrant autosomal dominant inheritance mechanism unlikely, but an X-linked recessive inheritance a plausible possibility given the preponderance of affected males in the study group.

### Mapping

As a first step, homozygosity mapping was used to identify the critical interval containing the causative variant. An initial group of nine ECS from Italy were genotyped using an Illumina Canine 220k SNP chip: two cases (ECS04 and ECS54), two unaffected parents (ECS05 and ECS06), one unaffected sibling (ECS07), two unaffected half-siblings (ECS55 and ECS36), and two unaffected related dogs (ECS37 and ECS48 - Fig. 2A, File S2). Assuming a monogenic recessive inheritance pattern, we searched for regions of homozygosity of chromosomal segments larger than 1 Mb identical by descent (IBD) in the affected dogs. Additionally, we considered the whole canine chromosome X (CFAX) as another plausible candidate interval due to the potential X-linked inheritance. Ultimately, the candidate regions fell within CFA4, CFA7, CFA10, CFA21, CFA29, and CFAX (Fig. 2B).

Once the critical autosomal homozygous candidate regions and chromosome X were confirmed, we converted the coordinates from Canfam 3.1 to the UU_Cfam_GSD_1.0 (canFam4, used for the Dog10k database). The entirety of the reported homozygous regions was counted as candidate intervals, as reported in Table 1. No other regions were shared by all the cases but not by the controls. Results for homozygosity mapping are shown in Fig. 2B, highlighting in red all the shared regions.

**Table 1:** Candidate intervals obtained through homozygosity mapping of affected and unaffected related dogs. Five autosomal homozygous candidate regions shared by the cases and not by the controls were identified, plus one putative candidate chromosome X additional interval. All coordinates are reported on the canFam4 reference

Chromosome	Start (bp)	End (bp)
chr4	31,342,838	34,222,026
chr7	41,518,928	42,442,001
chr10	13,529,131	70,640,185
chr21	18,879,226	30,510,868
chr29	16,521,663	21,420,081
chrX	1	124,992,030

### Variant detection and genotyping

Whole genome sequencing of a case (ECS04), his parents (ECS05 and 06), and another case (ECS54) was carried out. The reads were mapped against the UU_Cfam_GSD_1.0 reference (canFam4), and the called variants were filtered against the database. After filtering associated SNP or small indel variants, we next assessed the presence of large deletions, insertions, inversions, duplications, and translocations. The variants were once again filtered against internal controls generated by our lab for other studies, and the UU_Cfam_GSD_1.0 (canFam4) Dog10k database. Filtering assumed autosomal recessive inheritance (homozygous in both cases autosomes, heterozygous in the parents), and X-linked recessive inheritance (both parents unaffected, therefore variants missing in the father).

**Fig. 3:**
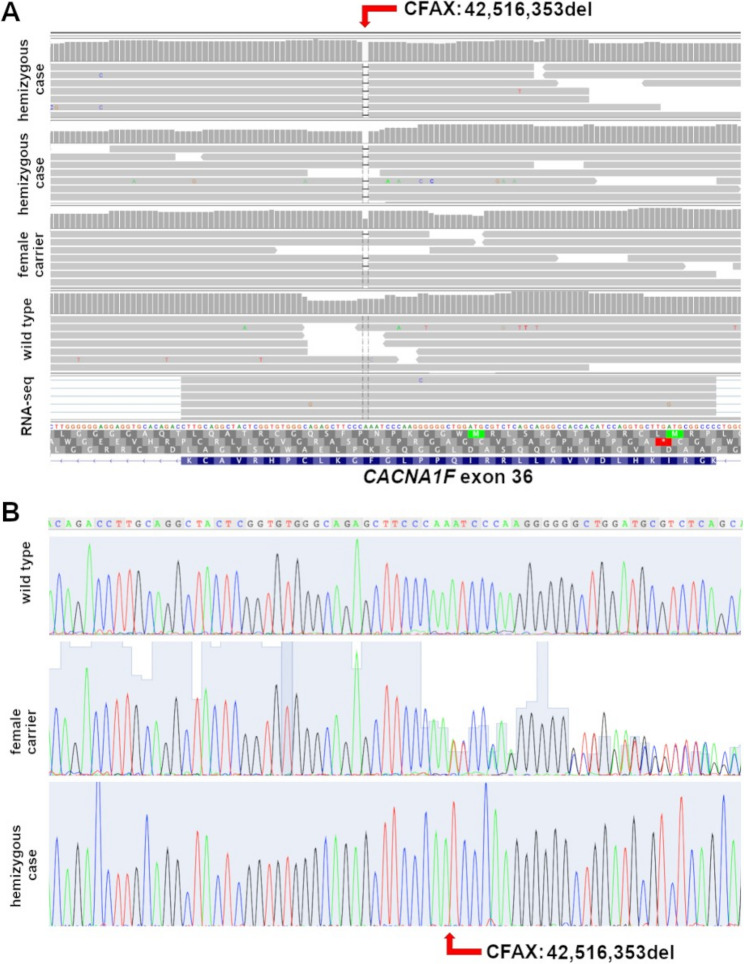
Sequencing of the putative causative variant, indicated with a red arrow. **A** Whole genome sequencing of two affected male cases (ECS04 and 54), the mother of ECS04 (ECS05, resulting as carrier), the father of ECS04 (ECS06, resulting as control), and of unrelated retinal RNA-seq (confirming the exon and transcript in retina), shown in Integrative Genomics Viewer. Note the 1-bp deletion (CFAX, 42,516,353del, canFam4 coordinates). **B** Sanger sequencing of a wild-type control (top), the unaffected female carrier (middle) and a hemizygous case (bottom) showing the deletion of the coding A

Following the filtering process, 285 SNVs and small indel variants remained as potential candidates. Of these, 284 were non-coding; one single striking high-impact coding variant was uniquely identified in our sequenced ECS, and was notably absent in all the control dogs sequenced in-house or present in the available databases. This variant resulted in a hemizygous 1-bp deletion in the coding region of the gene *CACNA1F* (exon 36), occurring in chromosome X in position 42,516,353 (UU_Cfam_GSD_1.0 NC_049260.1g.42,516,353del, Fig. 3). No filtered coding structural variants were detected within the candidate regions. Comparing the variant with the annotated transcript (XM_038587436.1, c.4,481del, https://www.ncbi.nlm.nih.gov/nuccore/XM_038587436.1, accessed 27 January 2026) and protein (XP_038443364.1 canine CACNA1F isoform X1 in NCBI, (https://www.ncbi.nlm.nih.gov/protein/XP_038443364.1, accessed 27 January 2026), we predicted that the protein would be affected by a frameshift and a premature stop codon (p.Phe1482LeufsTer8, File S3). This removes ~25% of the C-terminal region of the original protein.

As this variant resulted in a hemizygous frameshift in a retinal-expressed gene associated in humans with a retinal disease, we concluded that we had very likely identified the causative variant. Therefore, we designed a PCR test based on two primers to sequence the DNA of any available ECS cases and controls (Fig. 2A, Table 2, and File S4), and found co-segregation. A larger family tree has been built, showing the results obtained for all tested dogs and their relatedness (File S2).

**Table 2:** Distribution of the putative *CACNA1F* variant in our English Cocker Spaniel sample pool and in a database of canine variants (multiple breeds). It is to be noted that the genotyped carriers include the two mothers of all the cases, one female littermate of the cases, and one female ancestor on the maternal lineage. Both the fathers of the cases were clear, whereas one male, a common ancestor on the maternal side of all cases, was hemizygous, consistent with a recessive X-linked mode of inheritance. No homozygous affected females were detected. Two cases and two parents’ genotypes, as well as Dog10k database dogs’, were obtained by WGS – the others’ genotypes were obtained with Sanger sequencing

Breed and availability of retinal phenotype	*N*. of dogs	Genotype		
		wt/wt	wt/DEL	DEL
English Cocker Spaniel – Novel cases	4	0	0	4
English Cocker Spaniel – PRCD cases	5	5	0	0
English Cocker Spaniel – Unaffected	85	81	4	0
English Cocker Spaniel – Suspected cases*	1	0	0	1
Other breeds (Dog10k database)	1204	1204	0	0
Total	1299	1290	4	5

*Here we refer to ECS95, which is reported to be visually impaired by the owner and, coherently with the suggested transmission of the variant, resulted hemizygous affected

## Discussion

A combination of ancestry analysis, phenotyping, complementary testing for other known causative variants, homozygosity mapping, and whole-genome sequencing helped identify an X-linked high-impact variant in *CACNA1F* associated with XLPRA in a canine model and likely to be causative for the condition.

**Fig. 4:**
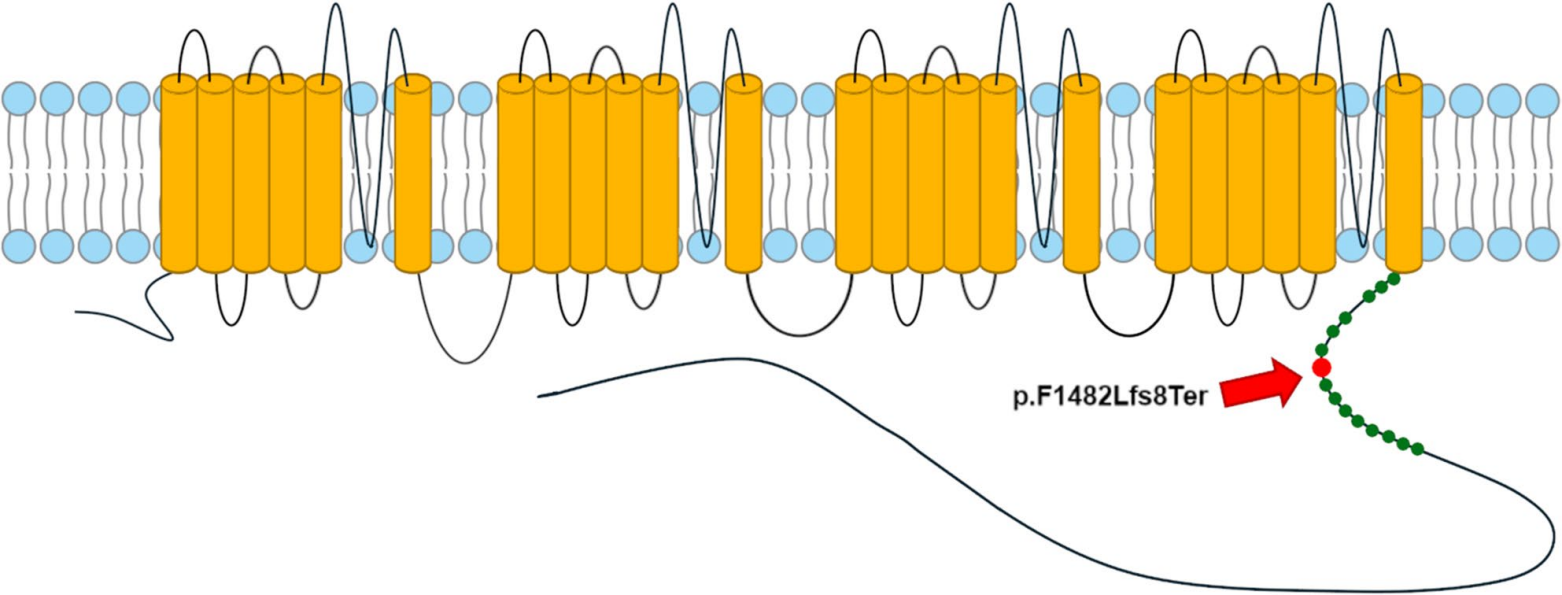
Schematics of the human CACNA1F protein. Cylinders represent the transmembrane domains. In the detail on the right, marked as a larger red dot, the position on the final C-terminal intramembrane chain of CACNA1F of our putative variant realigned against the human isoform. The smaller green dots represent the closest known causative variants for ocular diseases in the same chain. For a complete list, see File S5 - closest variants highlighted in yellow in the file. The protein topology is based on [46]

*CACNA1F* is a gene that codes for the Cav1.4 protein, an L-type Calcium channel. Ca^2^+ channels are predominantly expressed in retinal neurons, specifically at the level of the photoreceptor terminals [35]. There, they mediate the constant Ca2+ required for neurotransmitter release [35–37]. Genetic variants in *CACNA1F* are associated with ocular and retinal phenotypes in mammals; in humans, *CACNA1F* genetic variants are linked to three partially overlapping but distinct phenotypes, which all involve retinal manifestations (see OMIM 300071, 300476, and 300600, https://www.omim.org/entry/300110, accessed 8 September 2025). Variants in *CACNA1F* associated with type 2A incomplete congenital stationary night blindness have been identified in the 1990s [38, 39], with many more variants later mapped—see as an example Boycott et al., 2001 and Nakamura et al., 2003 [40, 41]. A recessive X-linked cone-rod dystrophy-3 (CORDX3) was mapped by Jalkanen [42]. Similarly, the Aland Island eye disease, described in 1964 [43] as a form of RP (earlier referred to as tapetoretinal degeneration, includes fundus hypopigmentation, decreased visual acuity, nystagmus, and astigmatism), was found associated with a homozygous 425-bp deletion in *CACNA1F* [44]. Murine models for *CACNA1F* knockouts have been generated, and a naturally occurring rat model has been identified [37]. Recently, a canine model has been identified in Greyhound, but the results have been published only in abstract form at the time of the completion of this manuscript [45]. A list of known variants in mammals is shown in File S5. Notably, intramembrane mutations close to the position of the canine variant we report are associated with retinal degeneration and myopia (Fig. 4, File S5).

The identification of a novel variant in the same gene known to cause retinal degeneration in humans underscores the shared genetic basis of disease across species. However, the impact of such findings extends beyond scientific relevance, highlighting critical considerations for breeding practices, veterinary screening, and the responsible use of genetic testing in companion animals.

Genetic tests are invaluable tools for monitoring known variants and reducing their prevalence in the population. However, a clear result on existing tests does not guarantee that a dog will not develop other genetic conditions. This is particularly relevant in the context of diseases with late onset, such as PRA. For these conditions, genetic testing should always be accompanied by regular veterinary screenings, which can enable the early detection of clinical signs, allow timely intervention, and guide reproduction.

Current breeding regulations also need to reflect this complexity. In Italy, for example, a dog can receive the title of “Selected Breeder” following appropriate genetic and veterinary evaluations. However, there is currently no requirement for continued screening to maintain this title. This means that a dog awarded the title at a young age could begin to show signs of PRA, cataracts or other inherited diseases later in life, yet still retain its breeding certification, potentially passing the condition on to offspring. Breeders and veterinarians should be actively encouraged to report suspected inherited diseases to researchers. Early reporting can enable a timely investigation into the genetic basis of such conditions, supporting the development of genetic tests to prevent further spread within the breed, to inform appropriate breeding strategies, and to assess frequency. This should probably be the aim of further studies, since this experimental design was specifically aimed at identifying the causative variant rather than estimating its prevalence in the breed population, thus not allowing conclusions on the frequency or geographic distribution of the variant within the ECS population.

All these considerations ultimately highlight the need for national kennel clubs and breed organizations to implement systematic biological sample collection and storage in biobanks accessible to researchers. Such infrastructures would be invaluable for identifying causative variants of emerging inherited conditions and for enabling informed genetic counselling for breeders and owners.

## Conclusions

The discovery of a novel variant in the *CACNA1F* gene associated with, and likely causative of, an X-linked form of progressive retinal atrophy in the English Cocker Spaniel adds a significant piece to the puzzle of inherited retinal diseases. Given the established role of this gene in similar disorders across multiple mammalian species, including humans, this finding reinforces its relevance in comparative ophthalmic genetics.

This result exemplifies the power of interdisciplinary collaboration—uniting breeders, veterinary clinicians, geneticists, and research institutions—in advancing our understanding of hereditary diseases. Indeed, detailed signalment of individuals affected by hereditary pathologies of unknown cause, coupled with comprehensive information on relatives and the collection of biological samples, can greatly accelerate the identification of novel variants. It also underscores the pivotal role that responsible breeders can play in scientific research, not merely as stakeholders but as active contributors to knowledge generation.

Such collaborative frameworks are essential to ensure that scientific progress translates into tangible benefits for animal health and welfare, such as the development of genetic tests for early detection of likely pathogenic variants, while also supporting informed and sustainable breeding practices.

## Supplementary Information

Supplementary Material 1. File S1. List of publicly available genomic data of internal controls. Bioproject, biosample and run ID are reported.

Supplementary Material 2. File S2. Complete pedigree of the sampled Italian ECS population. Squares represent males and circles represent females. Dogs subjected to genetic testing are shown in orange, whereas grey symbols indicate individuals without available genomic data. Female carriers are depicted with half-filled symbols, wild-type controls with empty symbols, and hemizygous males with symbols that are half filled and half diagonally barred.

Supplementary Material 3. File S3. Translated CACNA1F protein in wild-type and XM_038587436.1, c.4,481del variant. Starting and stop codons are marked in green and red, respectively.

Supplementary Material 4. File S4. Clinical phenotype, sex, and genetic testing results for sampled ECS dogs.

Supplementary Material 5. File S5. List of known *CACNA1F* variants in mammals. Variants shown in Figure 4 are highlighted in yellow.

## Data Availability

The datasets generated and/or analyzed during the current study are available in the Dryad repository, 10.5061/dryad.r7sqv9sqs.
