# Supplementary figures and images for: Deletion in CACNA1F gene causes X-linked progressive retinal atrophy in English Cocker Spaniel dogs
Source: BMC Vet Res. 2026 Mar 25;22:257. doi: 10.1186/s12917-026-05421-y (PMC13130813; doi:10.1186/s12917-026-05421-y)

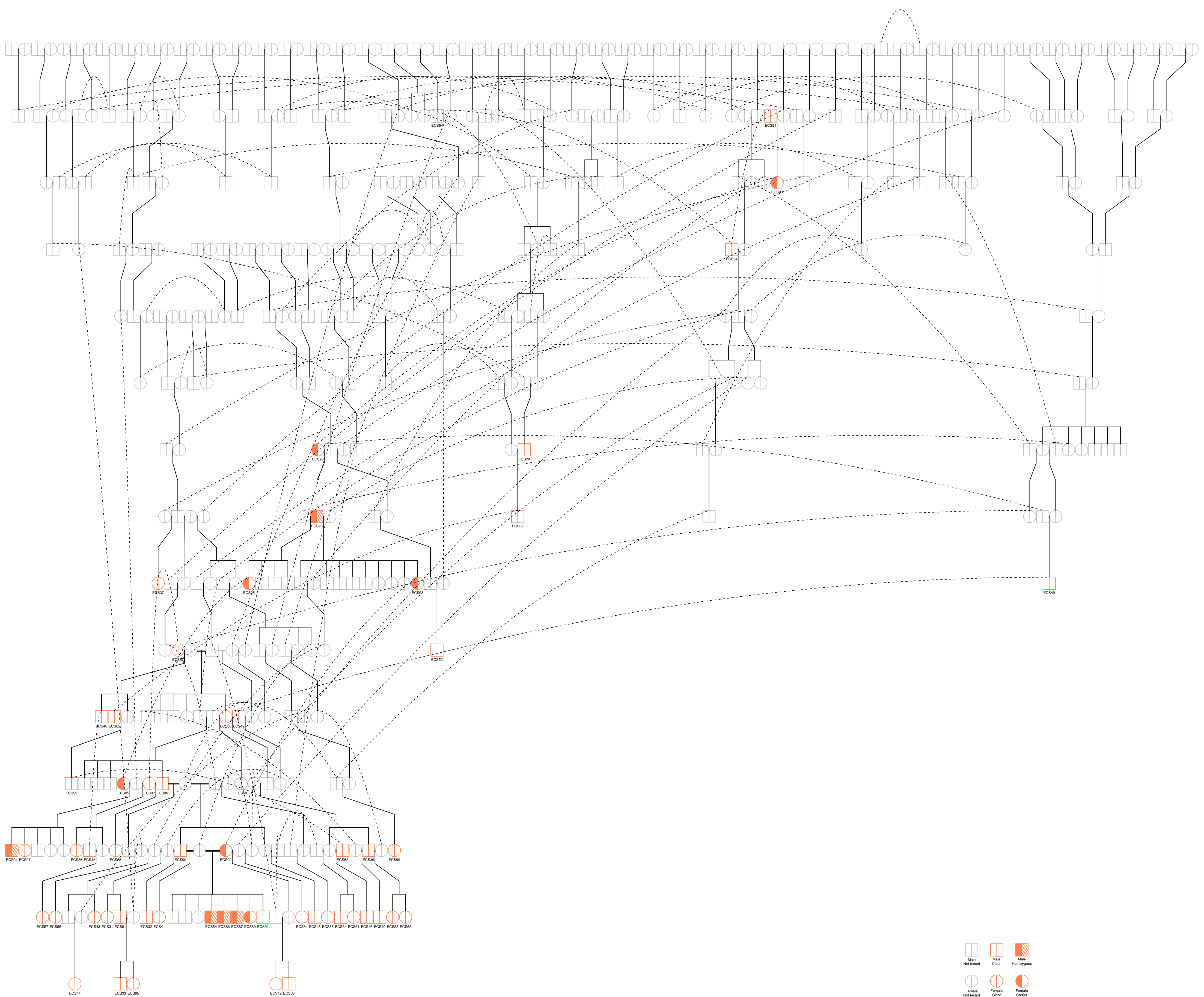

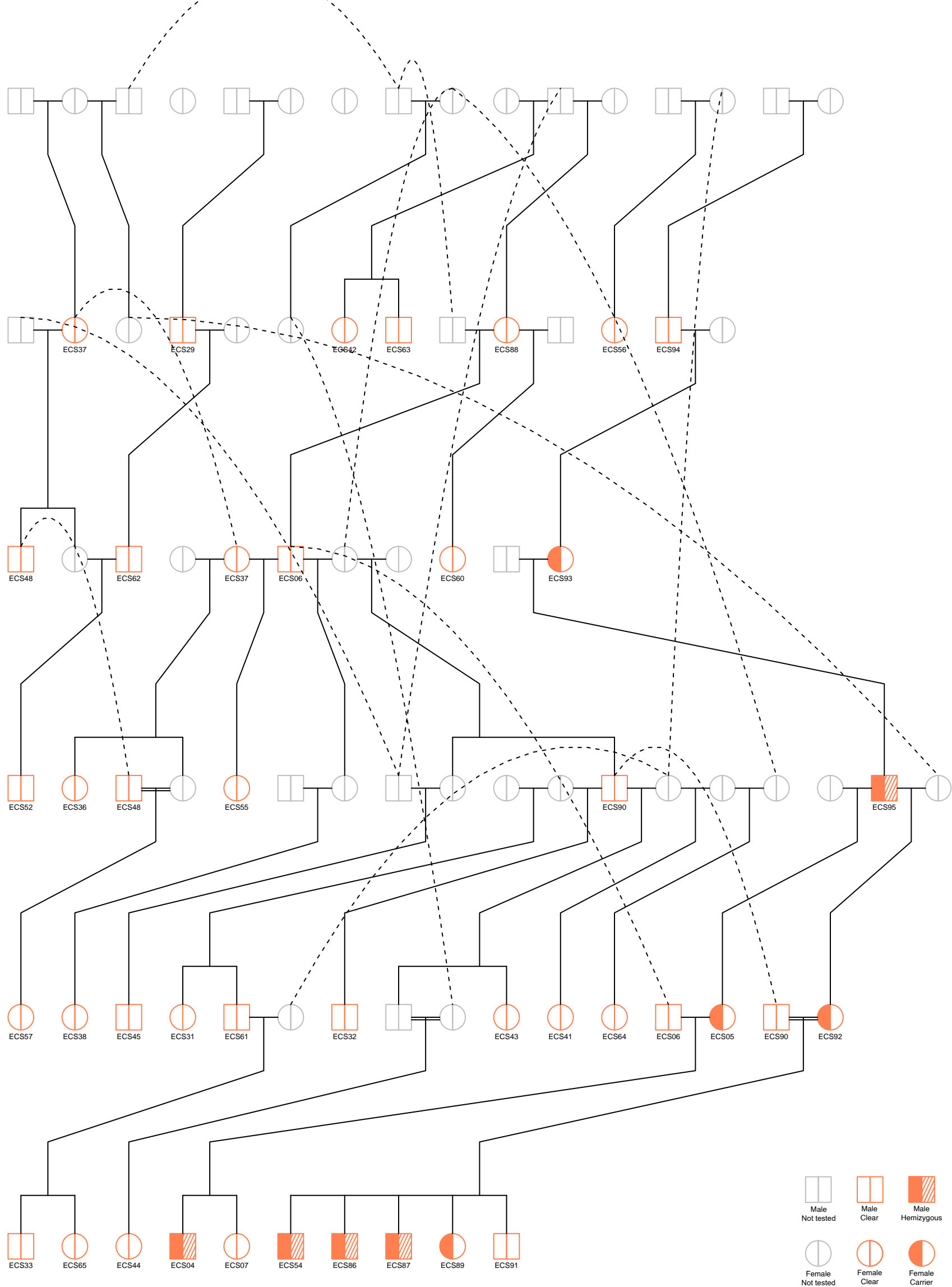

Supplement: Supplementary file 2 — Supplementary Material 2. File S2. Complete pedigree of the sampled Italian ECS population. Squares represent males and circles represent females. Dogs subjected to genetic testing are shown in orange, whereas grey symbols indicate individuals without available genomic data. Female carriers are depicted with half-filled symbols, wild-type controls with empty symbols, and hemizygous males with symbols that are half filled and half diagonally barred. [file 12917_2026_5421_MOESM2_ESM.pdf]
