# Supplementary material for: Deletion in CACNA1F gene causes X-linked progressive retinal atrophy in English Cocker Spaniel dogs
Source: BMC Vet Res. 2026 Mar 25;22:257. doi: 10.1186/s12917-026-05421-y (PMC13130813; doi:10.1186/s12917-026-05421-y)
Supplement: Supplementary file 3 — Supplementary Material 3. File S3. Translated CACNA1F protein in wild-type and XM_038587436.1, c.4,481del variant. Starting and stop codons are marked in green and red, respectively. [file 12917_2026_5421_MOESM3_ESM.docx]

>WT_Predict

MSASDGGKDTTPEPSPVNGTGPGPEWGLCPGPPASGGEEISGAVGPETPKRRTQHNKHKTVAVASAQRSPRALFCLTLANPLRRSCISIVEWKPFDILILLTIFANCVALGVYIPFPEDDSNTANHNLEQVEYVFLVIFTVETVLKIVAYGLVLHPSAYIRNGWNLLDFIIVVVGLFSVLLEQGPGRPGDTPHTGGKPGGFDVKALRAFRVLRPLRLVSGVPSLHIVLNSIMKALVPLLHIALLVLFVIIIYAIIGLELFLGRMHKTCYFLGSDLEAEEDPSPCASSGSGRACTLNQTECRGRWAGPNGGITNFDNFFFAMLTVFQCVTMEGWTDVLYWMQDAMGYELPWVYFVSLVIFGSFFVLNLVLGVLSGEFSKEREKAKARGDFQKLREKQQLEEDLRGYLDWITQAEELDLEDPSTDGNFGPQLADLTTRRRGHLRWFSHSTRSTHSTSSHASLPASDTGSMAEMPGDEEEEEGALASCTRCLNKIMKTRVCRRLRRVNRGLRARCRRAVKSTACYWAVLLLVFLNTLTIASEHHGQPMWLTQIQEYANKVLLCLFTVEMLLKLYGLGPSVYVSSFFNRFDCFVVCGGILETTLVEVGAMQPLGISVLRCVRLLRIFKVTRHWASLSNLVASLLNSMKSIASLLLLLFLFIIIFSLLGMQLFGGKFNFDQTHTKRSTFDTFPQALLTVFQILTGEDWNVVMYDGIMAYGGPFFPGMLVCVYFIILFICGNYILLNVFLAIAVDNLASGDTGTPKDKGKEKSTEGALPQENGVLVAGGENEEEEGRKNEGAGMEEEEEEEEGEEEEEGGAGHVELLQEVVPKEKVVPIPEGSAFFCLSQTNPLRKACHTLIHHHIFTNLILVFIILSSVSLAAEDPIRAHSFRNHILGYFDYAFTSIFTVEILLKMTVFGAFLHRGSFCRSWFNLLDLLVVSVSLISFGIHSSAISVVKILRVLRVLRPLRAINRAKGLKHVVQCVFVAIRTIGNIMIVTTLLQFMFACIGVQLFKGKFYSCTDEAKHTPQECKGSFLVYPDGDVSRPLVRERLWVNSDFNFDNVLSAMMALFTVSTFEGWPALLYKAIDANAEDKGPIYNYHVEISVFFIVYIIIIAFFMMNIFVGFVIITFRAQGEQEYQNCELDKNQRQCVEYALKAQPLRRYIPKNPHQYRVWATVNSAAFEYLMFLLILLNTVALAMQHYEQTAPFNYAMDILNMVFTGLFTIEMVLKIIAFKPKHYFTDAWNTFDALIVVGSVVDIAVTEVNNGGHLGESSEDSSRISITFFRLFRVMRLVKLLSKGEGIRTLLWTFIKSFQALPYVALLIAMIFFIYAVIGMQMFGKVALQDGTQINRNNNFQTFPQAVLLLFRCATGEAWQEIMLASLPGSRCDPESDVSPGEEFTCGSNFAIAYFISFFMLCAFLIINLFVAVIMDNFDYLTRDWSILGPHHLDEFKRIWSEYDPGAKGRIKHLDVVALLRRIQPPLGFGKLCPHRVACKRLVAMNMPLNSDGTVTFNATLFALVRTSLKIKTEGNLEQANQELRIVIKKIWKRMKQKLLDEVIPPADEEEVTVGKFYATFLIQDYFRKFRRRKEKGLLGSEAPSSTSSALQAGLRSLHDLGPEIRQALTCDTDEEEKEEEGLEGEEEEDEKHPETHKAQMGSQPPSRRSSVISVSLPVGDRLPDSLSLGPSDDDGGASNSRQSSVPQAGSHGHRRSSGGFIFTIPEEGSSQHKGTKEQENQEEEEEIPTKDSGHNRLSYLDEQAGTPPRPILLPPHRPQRCADGHNAPRRRLLPPTPAERKPSFTIQCLRRQGSCEDLPIPGTYHRGRNSGPSRAQGSWATPPQRGRLLYAPLLLVEEGAAGEGYLGKSSGPLRTFTCLRVPGTHSDSSHGKRGSADSLVEAVLISEGLGLFARDPRFVALAKQEIADACRLTLDEMDSAASDLLAQGTSSLYSDEESILSRFDEEDLGDEMACVHAL

>Mut_Predict

MSASDGGKDTTPEPSPVNGTGPGPEWGLCPGPPASGGEEISGAVGPETPKRRTQHNKHKTVAVASAQRSPRALFCLTLANPLRRSCISIVEWKPFDILILLTIFANCVALGVYIPFPEDDSNTANHNLEQVEYVFLVIFTVETVLKIVAYGLVLHPSAYIRNGWNLLDFIIVVVGLFSVLLEQGPGRPGDTPHTGGKPGGFDVKALRAFRVLRPLRLVSGVPSLHIVLNSIMKALVPLLHIALLVLFVIIIYAIIGLELFLGRMHKTCYFLGSDLEAEEDPSPCASSGSGRACTLNQTECRGRWAGPNGGITNFDNFFFAMLTVFQCVTMEGWTDVLYWMQDAMGYELPWVYFVSLVIFGSFFVLNLVLGVLSGEFSKEREKAKARGDFQKLREKQQLEEDLRGYLDWITQAEELDLEDPSTDGNFGPQLADLTTRRRGHLRWFSHSTRSTHSTSSHASLPASDTGSMAEMPGDEEEEEGALASCTRCLNKIMKTRVCRRLRRVNRGLRARCRRAVKSTACYWAVLLLVFLNTLTIASEHHGQPMWLTQIQEYANKVLLCLFTVEMLLKLYGLGPSVYVSSFFNRFDCFVVCGGILETTLVEVGAMQPLGISVLRCVRLLRIFKVTRHWASLSNLVASLLNSMKSIASLLLLLFLFIIIFSLLGMQLFGGKFNFDQTHTKRSTFDTFPQALLTVFQILTGEDWNVVMYDGIMAYGGPFFPGMLVCVYFIILFICGNYILLNVFLAIAVDNLASGDTGTPKDKGKEKSTEGALPQENGVLVAGGENEEEEGRKNEGAGMEEEEEEEEGEEEEEGGAGHVELLQEVVPKEKVVPIPEGSAFFCLSQTNPLRKACHTLIHHHIFTNLILVFIILSSVSLAAEDPIRAHSFRNHILGYFDYAFTSIFTVEILLKMTVFGAFLHRGSFCRSWFNLLDLLVVSVSLISFGIHSSAISVVKILRVLRVLRPLRAINRAKGLKHVVQCVFVAIRTIGNIMIVTTLLQFMFACIGVQLFKGKFYSCTDEAKHTPQECKGSFLVYPDGDVSRPLVRERLWVNSDFNFDNVLSAMMALFTVSTFEGWPALLYKAIDANAEDKGPIYNYHVEISVFFIVYIIIIAFFMMNIFVGFVIITFRAQGEQEYQNCELDKNQRQCVEYALKAQPLRRYIPKNPHQYRVWATVNSAAFEYLMFLLILLNTVALAMQHYEQTAPFNYAMDILNMVFTGLFTIEMVLKIIAFKPKHYFTDAWNTFDALIVVGSVVDIAVTEVNNGGHLGESSEDSSRISITFFRLFRVMRLVKLLSKGEGIRTLLWTFIKSFQALPYVALLIAMIFFIYAVIGMQMFGKVALQDGTQINRNNNFQTFPQAVLLLFRCATGEAWQEIMLASLPGSRCDPESDVSPGEEFTCGSNFAIAYFISFFMLCAFLIINLFVAVIMDNFDYLTRDWSILGPHHLDEFKRIWSEYDPGAKGRIKHLDVVALLRRIQPPLGLGSSAHTE
